# Unusual deletion of the maternal 11p15 allele in Beckwith–Wiedemann syndrome with an impact on both imprinting domains

**DOI:** 10.1186/s13148-021-01020-w

**Published:** 2021-02-04

**Authors:** Thomas Eggermann, Matthias Begemann, Lutz Pfeiffer

**Affiliations:** 1 Institute of Human Genetics, Medical Faculty, RWTH Aachen University, Pauwelsstr. 30, 52074 Aachen, Germany; 2 MVZ Medicover Humangenetik Berlin Lichtenberg, Berlin, Germany

**Keywords:** Beckwith–Wiedemann syndrome, 11p15.5 deletion, KCNQ1, IC2 hypomethylation, IGF2

## Abstract

**Background:**

Whereas duplications in 11p15.5 covering both imprinting centers (ICs) and their subordinated genes account for up to 1% of Beckwith–Wiedemann and Silver–Russell syndrome patients (BWS, SRS), the deletions in 11p15.5 reported so far only affect one of the ICs. In these cases, not only the size and gene content had an impact on the phenotype, but also the sex of the contributing parent influences the clinical signs of the deletion carrier.

**Results:**

We here report on the first case with a heterozygous deletion within the maternal allele affecting genes which are regulated by both ICs in 11p15.5 in a BWS patient, and describe the molecular and clinical consequences in case of its maternal or paternal inheritance.

**Conclusions:**

The identification of a unique deletion affecting both 11p15.5 imprinting domains in a BWS patient illustrates the complexity of the regulation mechanisms in these key imprinting regions.

## Background

The two differentially methylated imprinting control regions 1 and 2 (IC1, IC2) regulate the monoallelic and parent-of-origin dependent expression of a cluster of imprinted genes on chromosome 11p15.5. Whereas the paternally expressed *IGF2* and the maternally expressed *H19* genes are controlled by the telomeric and paternally methylated IC1 (*H19/IGF2*:IG-DMR), the maternally methylated and more centromeric IC2 (harboring the *KCNQ1OT1*:TSS-DMR) has an impact on the maternally expressed *CDKN1C* and the paternally expressed *KCNQ1OT1* genes.

Disturbances of the two ICs (aberrant methylation/epimutations of one of the two ICs; uniparental disomies and copy number variants of both ICs) are associated with two congenital imprinting disorders, Beckwith–Wiedemann and Silver–Russell syndrome (BWS: OMIM130650; SRS: OMIM180860). These two disorders show opposite molecular defects as well as opposite growth phenotypes. BWS is an overgrowth disorder with an increased risk of developing embryonal tumors and several birth defects (e.g., macroglossia, abdominal wall defects, neonatal hypoglycemia, lateralized overgrowth) (for review: [1]). Molecularly, the majority of patients exhibit a loss of methylation (LOM) of the IC2 (40%), followed by paternal uniparental disomies of 11p15.5 and gain of methylation (GOM) of the IC1. SRS is a growth retardation syndrome characterized by relative macrocephaly, a typical facial gestalt, asymmetry, feeding difficulties, and other less constant features (for review: [2]). In up to 40% of patients, a LOM of the IC1 can be observed. In addition to (epi)genetic disturbances of the IC1 and/or IC2, pathogenic variants within the *IGF2, CDKN1C* and *KCNQ1* genes have been reported in BWS and SRS (for review: [3–5]).

Deletions in 11p15.5 are rare [6–10], and losses affecting both imprinting regions have not yet been reported [8, 11–13]. In contrast, duplications of different sizes account for up to 1% of BWS and SRS patients, and they are either restricted to one of the two ICs, or affect both (for review: [13]).

We here report on the first case with a heterozygous deletion affecting the maternally inherited allele and genes regulated by both ICs in 11p15.5 in a BWS patient, and describe the molecular and clinical consequences in case of its maternal or paternal inheritance.

## Results

In a patient referred for routine molecular genetic testing for BWS, an unusual deletion within 11p15.5 was identified. The patient was born preterm to healthy non-related German parents at 36 weeks of gestation because of premature labor. Birth weight was 3820 g (2.15 z) [14], length 53 cm (1.5 z). A polyhydramnion was documented. Hypoglycemia was mentioned for first the 6 months of life, but treatment was not required. An omphalocele required five surgical interventions. Neuropsychomotor development was normal.

At the age of 30 years, the patient sought for genetic counselling. His height was 190 cm (1.37z), weight 110 kg (2.73z), and head circumference 58.8 cm (1.32 z). His left leg was 3 cm larger than the other. Pits were present at both ears. Clinical scoring on the basis of the recently consented system for BWS [1] resulted in a score of 9 points, supporting the clinical diagnosis of BWS (according to that system, a score of ≥ 4 points corresponds to BWS). Tumor monitoring until the seventh year of life was negative, as was cardiological surveillance at the age of 28 years.

Family history was empty, there was no history of assisted reproduction. The parents were of normal heights (mother 168 cm, father 175 cm), as was the healthy sister (172 cm).

**Fig. 1:**
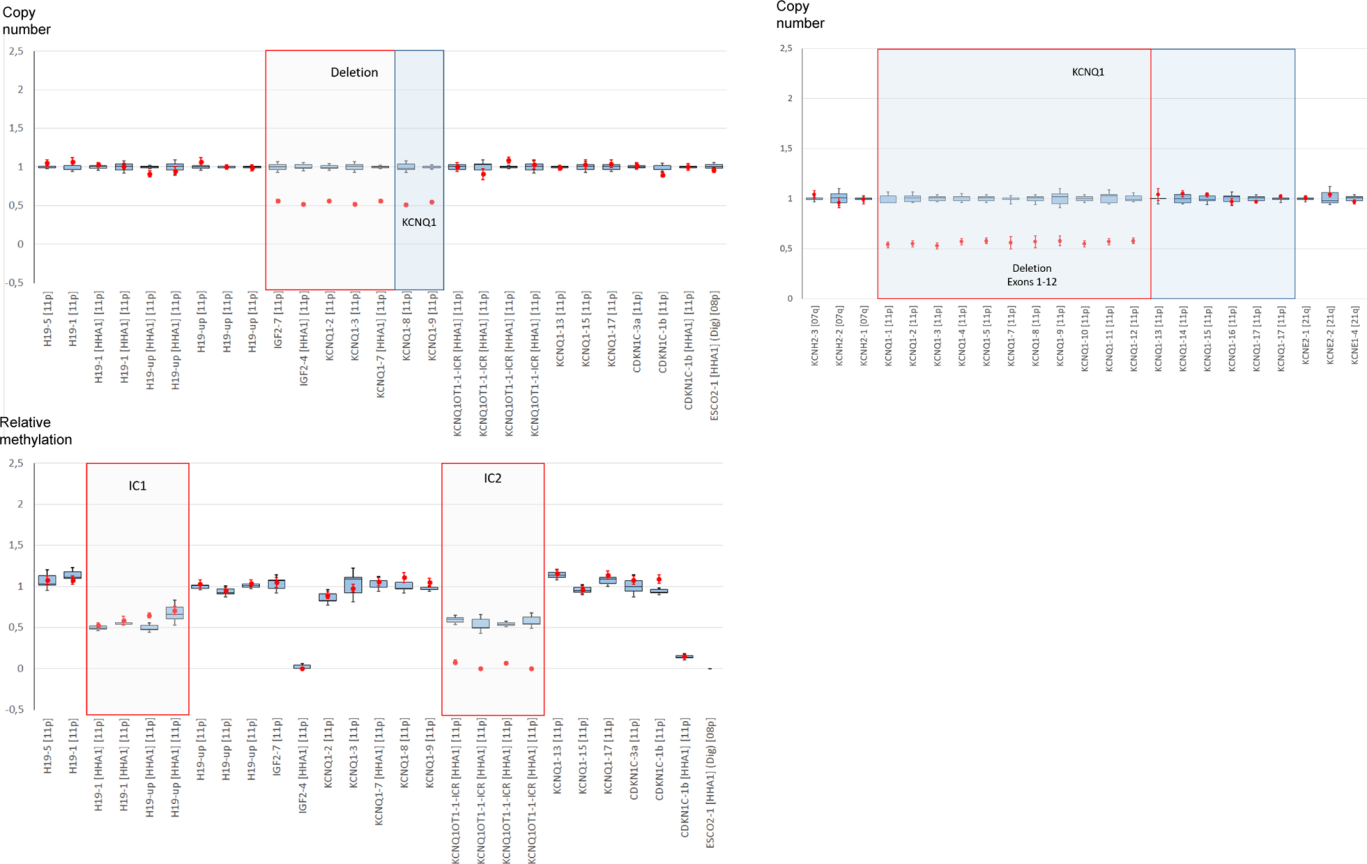
Identification of a deletion affecting the maternal *KCNQ1* allele by **a** methylation-specific MLPA analysis (assay ME030: upper panel: Copy number analysis, lower panel: methylation analysis) and **b** *KCNQ1*-specific MLPA (assay P114). The patient exhibits a deletion within the *KCNQ1* gene, but the IC2 sequence itself was not affected. Nevertheless, hypomethylation of the IC2 could be observed. (The control range was based on five individuals of normal epigenotype)

By methylation-specific multiplex ligation-dependent probe amplification assays (MS MLPA), a deletion affecting the *KCNQ1* gene and a loss of methylation of the IC2 was detected (Fig. 1), whereas methylation of the IC1 was normal. SNP array analysis revealed a size of 591 kb, (arr[hg19] 11p15.5(2125923_2716862) × 1)), including the whole *IGF2* gene, exons 1 to 12 of the *KCNQ1* gene (NM_000218.2), the whole *KCNQ1OT1* gene as well as further not imprinted genes (*INS, TH, ASCL2, C11orf21, TSPAN32, CD81, TSSC4, TRPM5*). In accordance with the MS MLPA results, neither the *KCNQ1OT1*:TSS-DMR nor the *H19/IGF2:*IG-DMR were affected.

MS-MLPA analyses of parental DNA samples targeting the 11p15.5 region gave normal copy number and methylation results.

## Discussion

**Fig. 2:**
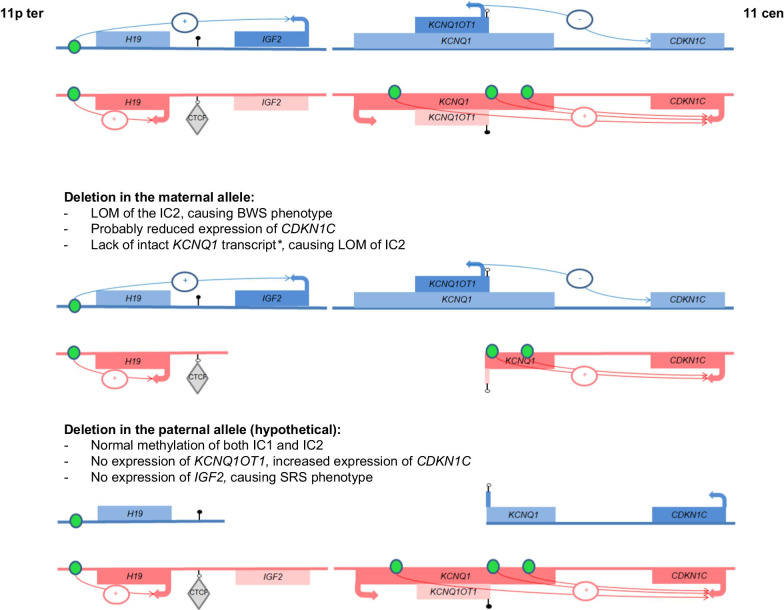
Simplified (hypothetical) effects of the deletion in our patient on the regulation of the imprinting domain in 11p15.5. In the upper figure, the normal situation is shown, whereas the effects of deletions in the maternal (corresponding to our patient) or in the paternal allele are illustrated and described in the lower figures. (*consequences for the *KCNQ1* isoform 2 which underlies genomic imprinting during embryogenesis; not to scale; arrows: expression of genes; filled lollipops: methylated ICs, empty lollipops: unmethylated ICs; green circles: enhancer elements; grey rhomb: CTCF; − suppression of expression, + enhancing of expression)

We report on the first deletion affecting parts of both imprinting domains in 11p15.5. Whereas recent reports describe patients in which only one of the two domains was affected and the disturbances had an impact on genes regulated by either the IC1 or the IC2, in our case, the disturbance of both regions has to be considered with respect to clinical significance and genetic counselling (Fig. 2).

In our patient, the 11p11.5 is associated with a loss of methylation of the maternally methylated IC2. It can therefore be concluded that the maternal 11p15.5 allele is affected, but that it probably occurs de-novo as molecular analysis of parental DNA samples gave normal results. As molecular alterations resulting in a IC2 loss of methylation are associated with BWS features, the phenotype in our patient is attributable to the disturbance of the centromeric imprinting domain. The deletion affects the *KCNQ1* and *KCNQ1OT1* genes, but the IC2 itself is not deleted. However, as several reports on pathogenic variants causing an aberrant *KCNQ1* transcript have already shown, only an intact *KCNQ1* transcript on the maternal chromosome can drive across the IC2, and is thereby the prerequisite for the IC2 de-novo methylation in the oocyte [5, 15, 16]. Accordingly, in case the deletion affects the maternal allele the IC2 remains hypomethylated, a molecular finding which is characteristic for BWS. For the transmission of the deletion via the paternal allele it can hypothesized that the variant does not alter the methylation status of the IC2 as the paternal allele is per-se not methylated.

The deletion also affects the noncoding RNA *KCNQ1OT1* which is transcribed only from the paternal allele, and suppresses the expression of the paternal *CDKN1C* copy, a negative regulator of cell proliferation. If *KCNQ1OT1* is partly deleted on the maternal allele, this should not impact *CDKN1C* expression as the maternal *KCNQ1OT1* is silenced whereas *CDKN1C* is expressed (Fig. 2). However, the deletion might affect *CDKN1C* expression as an enhancer motif for its expression has been suggested in this region [17]. Thus, a (slight) decrease of *CDKN1C* expression can be postulated and might contribute to the overgrowth phenotype in our patient. In case the deletion affects the paternal allele, expression of *KCNQ1OT1* is suppressed, and the overdose of *CDKN1C* results in a growth retardation phenotype [10].

Independent of the sex of the transmitting parent, the deletion of the first 12 exons of the *KCNQ1* gene predisposes for Long QT 1 syndrome. Therefore, carriers of truncating *KCNQ1* variants should be monitored cardiologically despite the variable penetrance of LQTS [18] which is confirmed by the negative cardiological examination results in our patient.

In the telomeric imprinting domain of our patient, the coding sequencing of the *IGF2* gene is deleted, but the *H19/IGF2*:IG-DMR is not affected and shows a normal methylation pattern.

As *IGF2* is transcribed from the paternal allele only, the deletion on the maternal chromosome 11p15.5 in our patient does not have an impact on his phenotype. The situation would change in case the paternal allele is affected, here the lack of *IGF2* should result in a growth retardation phenotype [3]. Children of our patient therefore have a chance of 50% to inherit the deletion and to be growth retarded due to the decreased expression of *IGF2* and increase of *CDKN1C* expression. The genomic sequence of *H19* gene is not affected by the deletion, but an alteration of its expression cannot be excluded as the chromatin structure of the region might be changed by the alteration. Finally, an altered expression of the other not imprinted genes within the deleted region cannot be precluded. However, evidences for their clinical relevance in patients with deletions or duplications in this region have not yet been reported.

## Conclusions

We report on a unique BWS patient with an alteration affecting both 11p15.5 imprinting domains, and thereby confirm the complexity of the regulation mechanisms in these key imprinting regions. Due to the different clinical consequences of 11p15.5 disturbances and the impact of the sex of the contributing parent, their precise size and genomic content has to be determined.

## Materials and methods

Genomic DNA of the patient was isolated from peripheral blood lymphocytes by simple salting out. Due to the BWS phenotype, the IC1 and IC2 in 11p15.5 were analyzed by two commercially available methylation-specific multiplex ligation-dependent probe amplification kits (MS MLPA) (ME030-C3 and ME034-A1, MRC Holland, Amsterdam/NL). The result was confirmed by another MLPA assay targeting the *KCNQ1* gene (assay P114-B3) (Fig. 1). Further characterization was conducted by SNP array analysis (CytoScan® HD Array (Affymetrix, Santa Clara/CA, USA)).

## Data Availability

The datasets used and/or analyzed during the current study are available from the corresponding author on reasonable request.

## Abbreviations

BWS: Beckwith–Wiedemann syndrome
IC1: Imprinting center region 1
IC2: Imprinting center region 2
GOM: Gain of methylation
LOM: Loss of methylation
MS MLPA: Methylation-specific multiplex ligation-dependent probe amplification
SRS: Silver–Russell syndrome
